# The Factorial Structure, Psychometric Properties and Sensitivity to Change of the Distress Tolerance Scale for Children with Emotional Disorders

**DOI:** 10.3390/children11010115

**Published:** 2024-01-17

**Authors:** Brígida Caiado, Diana Santos, Bárbara Pereira, Ana Carolina Góis, Maria Cristina Canavarro, Helena Moreira

**Affiliations:** Center for Research in Neuropsychology and Cognitive-Behavioral Intervention (CINEICC), Faculty of Psychology and Education Sciences, University of Coimbra, 3000-548 Coimbra, Portugal; uc2012155023@student.uc.pt (B.C.); uc2016218133@student.uc.pt (D.S.); uc2016227397@student.uc.pt (B.P.); uc2012132441@student.uc.pt (A.C.G.); mccanavarro@fpce.uc.pt (M.C.C.)

**Keywords:** distress tolerance, children, factor structure, psychometric properties, Portuguese

## Abstract

Background. The Distress Tolerance Scale (DTS) was adapted for American and Chinese youth, but never for European youth. Moreover, the factor structures found in these previous studies were not consistent. Methods. The DTS was adapted for Portuguese children and then validated among 153 children aged 6–13 years with emotional disorders. A confirmatory factor analysis (CFA) was conducted; the DTS reliability and validity were analyzed, and sex and age differences were explored. A sub-sample of children who received a transdiagnostic CBT (Unified Protocol for Children) was used to analyze the DTS’s sensitivity to therapeutic change. Results. The five tested models (based on previous studies) exhibited adequate fit in the CFA. However, the model previously reported for use in American children with emotional disorders was selected as the most appropriate. The DTS demonstrated adequate psychometric properties, and its validity was established through significant negative associations with measures of anxiety, depression and negative affect, as well as positive associations with positive affect. Age and sex differences were discussed. The DTS scores significantly increased from pre- to post-treatment, demonstrating sensitivity to therapeutic change. Conclusions. The DTS is a suitable and useful measure for assessing children’s distress tolerance and to assess the efficacy of CBT.

## 1. Introduction

Distress has been defined as a negative emotional state characterized by the tendency to alleviate the emotional experience [1]. On the other hand, distress tolerance refers to individuals’ ability to experience and endure negative and uncomfortable psychological states instead of avoiding them [1], as well as the capacity to persist in goal-directed tasks when faced with distress [2]. According to Simons and Gaher ([1], p. 83), distress tolerance “*consists of one’s evaluations and expectations of experiencing negative emotional states*” regarding (1) the perceived tolerability and aversiveness of distress; (2) the appraisal of distress and the acceptability of being in a negative emotional state; (3) the ability to not have one’s attention absorbed by this state, disrupting its function; and (4) the capacity to regulate emotions, controlling the tendencies to eliminate negative emotions [1].

According to this definition, individuals with low distress tolerance are less able to handle being distressed, and may feel ashamed of feeling distressed and therefore do not accept those feelings, perceiving their own coping skills as inferior to those of others. Their emotion regulation strategies are characterized by substantial effort to avoid unpleasant emotions and by the use of immediate methods to relieve the negative emotions experienced. When unable to avoid or alleviate negative emotional states, these individuals report being consumed by the experience, with their attention absorbed by the presence of upsetting emotions and their functioning significantly impaired [1].

Therefore, distress tolerance is often considered a meta-emotion construct, explaining the differences in predispositions to endure negative emotional states among individuals [3]. Accordingly, recent research has indicated that lower levels of distress tolerance can be considered an individual variable that contributes to the development and/or maintenance of a vast range of psychopathological symptoms and diagnoses (e.g., [4,5,6,7]), including anxiety and depressive symptoms and disorders [8,9]. As a result, several authors have identified distress tolerance as a transdiagnostic marker or risk factor for a wide range of psychopathologies [10,11,12], including emotional disorders, impacting both the intensity of experience and the use of coping strategies [13,14,15]. In fact, studies have shown that low distress tolerance significantly predicts the use of maladaptive emotion regulation strategies, including the suppression of emotions, avoidance, rumination and substance abuse [14,16,17], which implies that distress tolerance may influence emotion regulation styles [1]. This can happen because low levels of distress tolerance can be associated with more aversive reactions to emotions (i.e., not wanting to experience negative emotions), which in turn can lead to more emotional avoidance strategies [18]. Williams and colleagues [19], as well as McHugh and colleagues [20], have also proposed that because distress tolerance is a possible transdiagnostic vulnerability factor, it may be associated with changes in symptoms across treatment, which highlights the need to measure this construct throughout treatment. Additionally, some studies have highlighted the potential that distress tolerance is a moderating variable in the relationship between negative affect and the development of maladaptive symptoms and behaviors [21,22,23].

Research on distress tolerance among children and adolescents, particularly on its association with internalizing symptoms, is scarce. However, previous studies have shown that low distress tolerance is a predictor of more severe anxiety and depressive symptoms in nonclinical samples of children and adolescents [24,25,26,27], and that is associated with higher levels of externalizing problems (such as alcohol use and delinquent behaviors) among nonclinical samples of both Western and Chinese adolescents [28,29,30,31].

### 1.1. The Distress Tolerance Scale

Although distress tolerance has been the target of considerable research, the lack of self-report measures to assess this construct has been a limitation. Distress tolerance has been evaluated through broader related constructs, such as experiential avoidance [32,33] It has also been also frequently assessed through behavior analysis, particularly through persistence in the performance of physically or psychologically stressful tasks [2,34,35,36,37]. While these behavioral assessments have the advantage of being measurable outcomes, they could be influenced by the participant’s motivation to persist in the task or by the participants’ ability to tolerate pain or physical discomfort, both of which may differ from an individual’s tolerance for emotional discomfort [1].

With the aim of developing a quantitative measure to assess the perceived capacity to tolerate distress based on a multidimensional framework, Simons and Gaher [1] developed the Distress Tolerance Scale (DTS). The DTS is a 15-item self-report measure for adults, in which participants rate items on a 5-point Likert scale ranging from one (“strongly agree”) to five (“strongly disagree”). The items were developed based on their theoretical relevance as well as through a review of related scales. Using a factor analysis on a sample of 823 university students, the authors confirmed the four-factor structure of the DTS, with a higher-order factor of general distress tolerance [1]. The four factors were as follows: (1) tolerance, which assesses the ability to tolerate negative emotions; (2) appraisal, which assesses how an individual evaluates their negative emotions and how they perceive their own ability to tolerate them; (3) absorption, which assesses the extent to which individuals are controlled by their emotional distress and are consequently unable to complete other tasks; and (4) regulation, which assesses the efforts and strategies that individuals use to relieve distress and regulate themselves [1,38,39]. The DTS exhibited good psychometric properties in Simons and Gaher’s study [1], and when translated into several other languages, such as Persian [40], Turkish [41], French [42], Spanish [43,44], Pakistani [45], Polish [46] and Italian [3], as well as in several clinical samples, such as patients with obsessive compulsive disorders [47] and depression [48].

### 1.2. The Distress Tolerance Scale for Youths

Distress tolerance in children and adolescents has also mostly been assessed through behavioral measures [25,49]. To overcome this limitation, the psychometric properties of the DTS within samples of children and adolescents were analyzed by You and Leung [38], in a community sample of Chinese adolescents, and by Tonarely and Ehrenreich-May [50] in a clinical and community sample of American children and adolescents. Both studies confirmed that the DTS can be used as a 15-item measure of distress tolerance among youths. Nevertheless, the original factor structure suggested by Simons and Gaher [1] exhibited some alterations in these different age groups. This phenomenon could be explained by developmental effects, i.e., the ability to tolerate and regulate emotions in children and adolescents may differ from that of adults and from one another, since, for example, children may not yet have fully developed their cognitive and emotional skills. However, this type of reflection is still too early to draw, since there are few studies evaluating the age effect of age on distress tolerance and the results of studies with the DTS are not consistent regarding of its factorial structure found even within children and adolescents.

Specifically, in a community sample of 5423 Chinese adolescents aged between 12 and 19 years, You and Leung [38] confirmed the original four-factor structure (although Item 6 was moved from the appraisal to the regulation subscale). Nonetheless, contrary to previous studies with adult samples, the authors postulated that only three of the four first-order factors comprised the general distress tolerance factor: tolerance, appraisal and absorption [38]. The four subscales presented good internal consistency, with Cronbach’s alpha values above 0.70. The authors also examined sex differences in distress tolerance levels, and found that Chinese adolescent girls showed lower levels of distress tolerance and therefore were more likely to make immediate efforts to relieve distress than their male counterparts.

Tonarely and Ehrenreich-May [50] also examined the factor structure of the DTS in clinical (*n* = 165) and community (*n* = 117) samples of American children and adolescents (10–19 years old). Within the clinical sample, the factor structure was similar to that suggested by Simons and Gaher [1]. Nevertheless, similar to the findings of You and Leung [38], only tolerance, appraisal and absorption comprised the higher-order factor. The internal consistency of the four subscales and total score was adequate, with the exception of the appraisal subscale, which had a Cronbach’s alpha of 0.58. In the community sample, a three-factor structure (tolerance/appraisal, absorption and regulation) was found to be the best fitting model. The internal consistency of these subscales was adequate. Lower distress tolerance levels were found to be associated with more severe internalizing symptoms, which is consistent with the results of previous studies with both youth and adult samples (e.g., [1,25,29,51]). Additionally, the authors found that girls in the clinical sample presented lower levels of distress tolerance than males, and that older children in the community sample presented higher scores on the DTS.

### 1.3. Transdiagnostic CBT Intervention Aimed at Distress Tolerance

Transdiagnostic CBT interventions target the common processes or mechanisms underlying psychopathology (e.g., [52]). Since distress tolerance is a transdiagnostic mechanism (e.g., [20]), transdiagnostic interventions can be expected to increase levels of distress tolerance.

The Unified Protocol [52,53]) is a transdiagnostic CBT intervention for the transdiagnostic treatment of emotional disorders in adults. Through an emotion-focused approach, the Unified Protocol (UP) aims to reduce aversive and avoidant reactions to emotional experiences by reducing avoidance strategies that maintain psychopathology, and by reducing neuroticism [18,53,54]. This intervention was later adapted for adolescents and children, giving rise to the Unified Protocol for Children (UP-C) and the Unified Protocol for Adolescents (UP-A), respectively [53]. The UP-C consists of 15 weekly 90 min group sessions for children aged 6 to 12 years old with emotional disorders and their parents. This intervention uses psychoeducation, cognitive, problem-solving training, present-moment and non-judgmental awareness and situational emotional exposure techniques. The UP-C has already demonstrated its efficacy in treating children’s emotional disorders within American children and their parents [48], and feasibility studies have been conducted in Portugal [55] and Japan [56], confirming that the UP-C was viable and accepted by Portuguese and Japanese populations. Currently, the efficacy of this intervention for treating emotional disorders in Portuguese children is under investigation (ClinicalTrials.gov NCT04932421). Although there are still few efficacy studies with the UP-C and none assessing the intervention’s effect on promoting transdiagnostic mechanisms, such as distress tolerance, it is expected that the UP-C, because of the type of strategies it uses and because it adopts an emotion-centered approach, may promote a greater ability to identify, regulate and tolerate negative emotions, and therefore may increase distress tolerance.

### 1.4. The Present Study

To the best of our knowledge, the factor structure and psychometric properties of the DTS in youths have been analyzed in only Chinese and American populations. Additionally, the factor structures found in these studies were not consistent, and only Tonarely and Ehrenreich-May [50] used a clinical sample. Therefore, given the relevance of the assessment of distress tolerance in youths for understanding and treating psychopathology in children, it is important to evaluate the psychometric properties of the DTS in other youth populations; namely, the European population, particularly within clinical samples. Furthermore, there is no measure of distress tolerance available for the Portuguese population, which limits empirical investigations using this critical construct. For these reasons, the present study aimed to adapt the DTS for use in Portuguese children and to assess its factor structure and psychometric properties in a clinical sample. The specific objectives of this study are shown below.

Aim 1. To analyze the factor structure of the DTS and its adequacy within a sample of Portuguese children with an emotional disorder, we examined five competing models based on previous studies: (1) the hierarchal model proposed by Simons and Gaher [1], based on a nonclinical sample of adults; (2) a model similar to that proposed by Simons and Gaher, but a four-dimensional instead of an hierarchical model; (3) the model proposed by You and Leung [38], based on a nonclinical sample of adolescents; (3) the model proposed by Tonarely and Ehrenreich-May, based on a nonclinical sample of children; and (4) the model proposed by Tonarely and Ehrenreich-May, based on a clinical sample of children. We expected to find results similar to those of Tonarely and Ehrenreich-May in the clinical sample of children due to the similarity of the sample in the present study.

Aim 2. To evaluate the internal consistency of the DTS and to explore its validity through its correlation with measures of anxiety, depression, and negative affect. Based on previous research showing that lower levels of distress tolerance were predictors of more severe anxiety and depressive symptoms in youths (e.g., [25,28,29]), we expected higher scores of distress tolerance to be positively correlated with positive affect and negatively correlated with anxiety, depression and negative affect.

Aim 3. To assess whether DTS scores are significantly correlated with sex and age.

Aim 4. To analyze to what extent the DTS is sensitive to therapeutic change. Although distress tolerance has been typically analyzed as a predictor of treatment, we expect that CBT intervention (based on psychoeducation to normalize and explain the function of emotions, present moment awareness strategies, and emotional exposure) will improve levels of distress tolerance as assessed by the DTS.

## 2. Methods

### 2.1. Procedure

#### Translation Process

We obtained authorization from the authors of the original version to translate the DTS into Portugues and analyze its psychometric properties. The DTS items were then independently translated by two Portuguese researchers. The first Portuguese version was developed after the comparison and discussion of the similarities and differences between the two translations. The translation of the terms “*distressed* or *upset*” were the most challenging and led to the most discussion among translators, since the literal translation of the word “*upset*” (i.e., “*chateado*”) does not seem to represent the concept of “*distress tolerance*”, since “*chateado*” has a connotation more similar to that of the English word “*angry*”. Indeed, the word “*upset*” could be translated as *“chateado*” (angry), “*frustrado*” (frustrated), “*nervoso*” (nervous) or “*perturbado*” (disturbed). To overcome this issue, the translators agreed upon the translation of “*upset*” into “*chateado ou frustrado*” (i.e., upset or frustrated).

Then, the DTS was administered to a group of seven children aged 6–11 years (*M* = 8.00; *SD* = 1.92) with emotional disorders. This revealed difficulties in children’s understanding of the scale, which led the researchers who administered the scale to believe that this translation did not provide an accurate assessment of “*distress tolerance*” as the ability to experience and endure negative and uncomfortable psychological states, rather than avoiding them [1]. Consequently, the translation was revised, and the original instruction “*think of times that you feel distressed or upset*” was changed to *“think of times that you feel negative emotions (e.g., very nervous, anxious, irritable, worried, or sad)”*. In addition, the expression “*feeling distressed or upset*” was replaced by “*feeling negative emotions*” in all items. The translators felt that these modifications would improve children’s understanding of the DTS and better convey the concept of “*distress tolerance*”.

In addition, although the original scale (developed for use in adults) used a 5-point Likert scale from 1 = “strongly agree” to 5 = “strongly disagree”, this type of scale was confusing for young children in our 7-child sample. Therefore, to make it easier for young children to understand, we adopted an inverse scale where 1 = “strongly disagree” and 5 = “strongly agree”, which was more intuitive for children. However, to maintain the same rating as the original scale (in which higher scores represent higher levels of distress tolerance), the item scores were reversed for analysis.

This final version (Appendix A) was administered to 25 children aged 6–12 years (*M* = 8.32; *SD* = 1.84) with emotional disorders, and after a brief cognitive debriefing, the instructions and items were found to be clear.

### 2.2. Participants

A total of 153 children from central Portugal with a mean age of 9.44 years (*SD* = 1.83, range = 6–13) and with a primary diagnosis of an emotional disorder (92.2% with an anxiety or anxiety-related disorder; 7.8% with depression) were recruited. Further information about their sociodemographic and clinical characteristics is presented in Table 1.

### 2.3. Procedure

Data were collected as part of a randomized controlled trial aiming to assess the efficacy of the Unified Protocol for the Transdiagnostic Treatment of Emotional Disorders in Children (UP-C; [11]) among Portuguese children with emotional disorders. The UP-C is composed of 15 weekly group sessions that provide psychoeducation about emotions to raise awareness and normalize them, practicing present moment awareness and non-judgmental awareness, and facing situations that trigger intense emotions through emotional exposures.

Participants were referred by mental health professionals from of a central hospital collaborating in the study (Centro Hospitalar Tondela-Viseu), school psychologists from collaborating schools, or recruited through parents’ self-registration on the project website. To be eligible for the study, children had to be aged between 6 and 13 years and have an emotional disorder as their primary diagnosis (i.e., anxiety or depressive disorders). After an initial eligibility interview, in which a diagnostic clinical interview based on the DSM-5 was used, children were randomly assigned to one of two study conditions: the experimental group (UP-C) and the control group (a psychoeducation intervention). The data used in the present study are from the pretreatment assessment. In the analysis of sensitivity to change, post-treatment data from the experimental group was also included. Information regarding the items belonging to each subscale and their Portuguese translation can be found in Table 2.

### 2.4. Measures

#### 2.4.1. Distress Tolerance

The Distress Tolerance Scale [1], a 15-item self-report measure that assesses how individuals perceive their own ability to handle aversive emotions, was translated into Portuguese and adapted for use in children. In the DTS, children report how much they agree with a given statement on a 5-point Likert scale, ranging from 1 (“*strongly disagree*”) to 5 (“*strongly agree*”). The DTS has four subscales: (1) tolerance, which assesses the ability to tolerate negative emotions (e.g., “*I can’t handle feeling negative emotions”)*; (2) appraisal, which assesses how the children evaluate their negative emotions and how they perceive their own ability to tolerate them (e.g., “*I am ashamed of myself when I feel negative emotions*”); (3) absorption, which assesses the extent to which their emotional distress prevents children from completing other tasks (e.g., “*When I feel negative emotions, all I can think about is how bad I feel*”); and (4) regulation, which assesses the efforts to relieve distress (e.g., “*I’ll do anything to avoid feeling negative emotions*”). The total score for the subscales was calculated as the mean of the items corresponding to each subscale. All items, except item 6, were reserved; therefore, higher scores indicate higher levels of distress tolerance.

#### 2.4.2. Anxiety and Depression Symptoms

The Revised Child Anxiety and Depression Scale (RCADS; [57,58]) is a 47-item self-report scale that assesses children’s anxiety and depression symptoms on a 4-point Likert scale ranging from 0 (“*never”*) to 3 (“*always”*). The RCADS has a total internalizing score (sum of all items), a total anxiety score (sum of items on the anxiety subscales; 37 items) and a total depression score (sum of the items belonging to the depression subscale; 10 items; e.g., “*I feel sad or empty*”). The anxiety scale is made up of five subscales that assess symptoms associated with anxiety disorders in the DSM-5 (namely, separation anxiety disorder, generalized anxiety disorder, panic disorder, social phobia and obsessive compulsive disorder; e.g., “*I worry that something bad will happen to me*” and “*I worry what other people think of me*”). Higher scores indicate more severe anxiety and/or depression symptoms. In the current study, Cronbach’s alpha coefficients were 0.94 for the total scale, 0.93 for the anxiety scale and 0.84 for the depression scale.

#### 2.4.3. Positive and Negative Affect

The Positive and Negative Affect Schedule for Children-Short Form (PANAS-C-SF; [59,60]) is a 10-item self-report scale that examines children’s positive and negative affect. Children are asked to indicate the frequency with which they experience a range of feelings on a 5-point Likert scale ranging from 1 (*very slightly or never*) to 5 (*very much*). The PANAS-C-SF has two scales: the positive affect scale, composed of 5 items (for example, “*happy”*), and the negative affect scale, also composed of 5 items (for example, “*scared”*). Higher levels of positive or negative affect are indicated by higher scores on these scales. In the current study, Cronbach’s alpha coefficients were 0.78 for the negative affect scale and 0.87 for the positive affect scale.

### 2.5. Statistical Analyses

SPSS version 26.0 (IBM SPSS, Chicago, IL, USA) was used to compute descriptive statistics regarding the participants’ sociodemographic characteristics.

**Figure 1:**
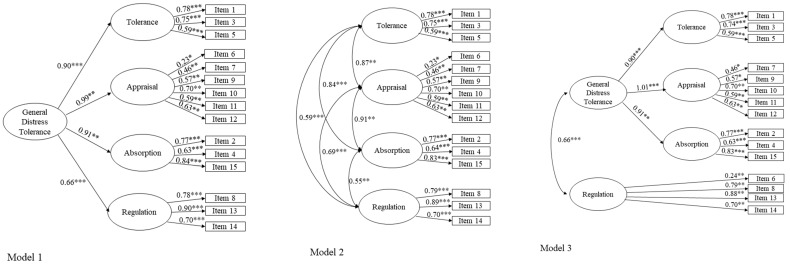

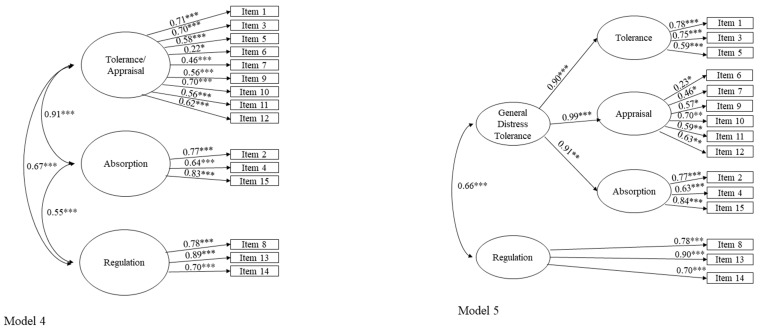
CFA Models. Note. * *p* < 0.05; ** *p* < 0.01; *** *p* < 0.001; Model 1: Simons and Gaher’s [1] model for non-clinical adult sample; Model 2: A four-dimensional based on Simons and Gaher’s [1] model; Model 3: You and Leung’s [38] model for non-clinical adolescent sample; Model 4: Tonarely and Ehrenreich-May’s [50] model for non-clinical children and adolescent sample; Model 5: Tonarely and Ehrenreich-May’s [50] model for clinical children and adolescent sample.

To assess and compare the model fit of the five models (see Figure 1), a confirmatory factor analysis (CFA) was conducted using AMOS (IBM^®^ SPSS^®^ AMOS™ version 24.0; IBM Corporation, Meadville, PA, USA). The comparative fit index (CFI), the root mean square error of approximation (RMSEA) and the normalized root mean square residual (SRMR) values were used to determine model fit using the following criteria: CFI ≥0.90, Tucker–Lewis index (TLI) ≥0.90, RMSEA ≤0.08 and SRMR ≤0.06 [61,62]. Factor loadings ≥ 0.32 were considered significant [63] Akaike’s information criterion (AIC), a relative measure of the parsimony of models, was used to compare models, with a lower AIC denoting a more parsimonious model [64]. Nested models were compared using the chi-squared difference test.

Cronbach’s alpha coefficients for the DTS were also calculated using SPSS, with values above 0.70 indicating good reliability [65]. The item-total correlation and the value of Cronbach’s alpha coefficients if item is deleted were also computed. The item-total correlation should be greater than 0.30 [66].

Pearson correlation analyses of the relationships between the DTS subscales and between the DTS subscales and children’s anxiety and depression levels (RCADS scores) as well as positive and negative affectivity (PANAS-C-SF scores) were conducted using SPSS to investigate the validity of the DTS.

To examine the associations of distress tolerance with sex and age, Pearson correlation analyses were performed using SPSS.

## 3. Results

### 3.1. The Factorial Structure of the DTS—Confirmatory Factor Analysis (Aim 1)

The model fit results of the CFA are presented in Table 3. All five models presented an adequate fit to the data. As presented in Figure 1, in all models, all paths were statistically significant, and the majority of factor loadings were above 0.32, except for Item 6. Therefore, all models were tested without Item 6 to assess whether its removal would improve the model fit; however, the results were very similar to those when including Item 6, with no significant difference [Model 1 and Model 2—Δχ^2^ (13) =21.6, *p* = 0.06; Model 3—Δχ^2^ (13) = 20.9, *p* = 0.07; Model 4—Δχ^2^ (13) = 21.9, *p* = 0.06; Model 5—Δχ^2^ (13) = 21.6, *p* = 0.06]; therefore, we decided to retain Item 6.

Regarding comparisons among the analyzed models, all had very similar results. Comparing the AIC values, Model 3 and Model 5 seemed to be the best fitting models. Given its similarity, we decided to select Model 5 because it was studied in a clinical sample of children with emotional disorders, which is most similar to the present sample.

### 3.2. Internal Consistency (Aim 2)

As described in Table 2, the DTS total score and its subscale scores presented good reliability values, as indicated by Cronbach’s alpha values equal to or greater than 0.70. According to the item-total correlation, all items seemed to contribute favorably to the scale, with values above 0.30, except for Item 6, which had item-total correlation of 0.22. Moreover, based on the Cronbach’s alpha value if item is deleted, the large majority of items contributed favorably to the scale, except for Items 4, 5 and 6, which, if removed, slightly increased the alpha value of the respective subscale (from 0.78 to 0.80, from 0.73 to 0.76 and from 0.70 to 0.72, respectively).

### 3.3. Correlations between DTS Scores and Measures of Anxiety, Depression and Negative Affect (Aim 2)

As presented in Table 4, the correlations of the DTS total score and the DTS subscale scores with measures of anxiety, depression and negative affect were negative and statistically significant. The correlations of the DTS total score and the DTS subscale scores with positive affect were positive and statistically significant. However, the regulation subscale presented a nonsignificant correlation with positive affect.

### 3.4. Correlations among DTS, Sex and Age (Aim 3)

The DTS total score and the DTS subscale scores were not significantly correlated with the children’s age (Table 5). Regarding sex (coded as 0 for boys and 1 for girls), there were statistically significant correlations with only the DTS total score and the appraisal subscale score, with boys presenting higher distress tolerance scores (DTS total scale) than girls and higher appraisal scores than girls (see Table 5).

### 3.5. Sensitivity to Therapeutic Change (Aim 4)

As presented in Table 6, the DTS mean scores increased significantly from pretreatment to post-treatment, indicating sensitivity to change, with a large effect size.

The DTS scores post-treatment also presented good internal consistency, with Cronbach’s alpha values of 0.93 for the total scale, 84 for the tolerance subscale, 0.82 for the regulation subscale, 0.82 for the absorption subscale and 0.81 for the appraisal subscale.

## 4. Discussion

Although distress tolerance is considered an important variable in the understanding and treatment of children’s and adults’ psychopathology, the lack of adequate measures to assess this construct has limited the development of empirical studies in this area. To fill this gap, Simons and Gaher [1] developed the DTS, a self-report measure to assess distress tolerance in adults. Due to the relevance of this construct in youths, the DTS was later adapted for use in clinical and nonclinical samples of children and adolescents in Chinese and American populations (e.g., [38,50]). The present study aimed to translate and adapt the DTS for use in the Portuguese population and to evaluate its psychometric properties in a sample of children with emotional disorders.

In this study, the DTS was first translated into Portuguese. The initial Portuguese version of the DTS was administered to a group of seven children aged 7 to 11 years with emotional disorders. Subsequently, several revisions were made to ensure that the scale was comprehensible for Portuguese children. The final version of the translation was administered to 25 children aged 7 to 12 years, and proved to be adequately understandable. Therefore, the DTS was administered to 153 children aged 6 to 13 years who were diagnosed with emotional disorders to assess its psychometric properties. Four main objectives guided this study:

Aim 1. The five competing models reflecting different factor structures found in previous studies were analyzed through CFA and compared [Model 1 was proposed by Simons and Gaher [1], based on a nonclinical sample of adults; Model 2 was a four-dimensional model based on Model 1; Model 3 was proposed by You and Leung [38], based on a nonclinical sample of adolescents; Model 4 was proposed by Tonarely and Ehrenreich-May [50], based on a nonclinical sample of children; and Model 5 was also proposed by Tonarely and Ehrenreich-May [50], based on a clinical sample of children]. The five analyzed models presented adequate model fitting to the data. After comparing model fit and taking into account the characteristics of the population in which they were developed, Model 5 was retained. This decision was based on the fact that Model 3 and Model 5 were the best fitting models, and, considering the similarities in terms of fit between the two models, Model 5 was retained because it was studied in a population more similar to that of the present study (i.e., children with emotional disorders).

The two models with the best fit (Model 3 and Model 5) were both composed of a general factor of distress tolerance (which includes the sub-scales of tolerance, appraisal, and absorption) and a general factor of regulation. This means that, according to these models, in the sample of the present study, regulation was not considered part of distress tolerance as a general factor, even though they are significantly and positively correlated with each other (i.e., the greater the ability to tolerate one’s emotions, the greater the ability to regulate them and to not make any effort to rid oneself of them). Therefore, distress tolerance as a general factor appears to be more related to more cognitive dimensions and perceived emotional ability (i.e., the ability to tolerate one’s emotions; how a child appraises their negative emotions and their ability to tolerate them; and how the child feels consumed or absorbed by their negative emotions). On the other hand, the efforts that a child makes to rid themselves of their negative emotions (regulation scale—more related to adopted behaviors), although closely related to the construct of distress tolerance, seems to be better understood as a separate general factor. Therefore, these results seem to have theoretical implications for the understanding of distress tolerance in children aged 6 to 12 years old.

Throughout the statistical analyses, Item 6 demonstrated some psychometric weaknesses. In the CFA, the path related to Item 6 was significant (demonstrating the relevance of this item to the model), but the loading factor was lower than that recommended in the literature (i.e., lower than 0.32). Moreover, Item 6 also presented an item-total correlation below the recommended value (i.e., 0.30), and the Cronbach’s alpha value of the subscale to which Item 6 belongs increased from 0.70 to 0.72 after removing Item 6. Due to these weaknesses, the four tested models were retested without Item 6; however, the difference in model fit with and without Item 6 was not significant, so we decided to retain Item 6.

Additionally, Items 4 and 5 were also found to lower the reliability of the subscales to which they belonged. Specifically, if Items 4 and 5 were removed, the alpha values of the respective subscales would slightly increase from 0.78 to 0.80, and from 0.73 to 0.76, respectively. However, since the paths of these items were significant, they had factor loadings greater than 0.32 (in the CFA), and the item-total correlation was greater than 0.30; as recommended, we decided to keep these items.

Aim 2. The internal consistency of the DTS was analyzed, and, as expected, adequate to good reliability values were obtained (i.e., Cronbach’s alpha values equal to or greater than 0.70 for all subscales and for the total score). Additionally, and as expected, higher levels of distress tolerance were significantly associated with higher levels of positive affect and with lower levels of negative affect, anxiety symptoms and depressive symptoms. These results applied to both the DTS total score and its subscale scores, except for the regulation subscale. Although this subscale was positively associated with high levels of positive affect, the association was not significant. Globally, these results are in line with our hypotheses and with those postulated in previous studies (e.g., [25,28,29]). Children with low distress tolerance are likely to struggle more in managing their negative emotions and are more prone to engage in maladaptive emotional regulation strategies to avoid experiencing unpleasant emotions [1,14,16,17]. In turn, the engagement in such maladaptive emotional regulation strategies can predispose and perpetuate psychopathological symptoms, particularly those related to anxiety and depression [8,9,25] which in turn sustains high levels of negative affect and low levels of positive affect. On the other hand, since children with low distress tolerance have greater difficulty in tolerating negative emotions [1], it is expected that children with higher levels of negative affect (i.e., a greater tendency to experience negative emotions) will have lower distress tolerance, and those with higher levels of positive affect (i.e., a greater tendency to experience positive emotions) will have higher distress tolerance. Concurrently, children with depression and anxiety exhibit higher levels of negative affect and lower levels of positive affect [67]; hence, these children are more likely to have low distress tolerance Thus, the results found in this study support the validity of the DTS.

Aim 3. The associations of distress tolerance (DTS scores) with sex and age were also explored. In the present study, no significant associations were found between levels of distress tolerance and age, which means that younger and older children seem to exhibit similar levels of tolerance for negative emotions. These results are congruent with previous studies (e.g., [50]), supporting the idea that distress tolerance is stable across age during childhood.

Regarding sex, boys appeared to have slightly higher tolerance for negative emotions (DTS total score) than girls, albeit with a small effect size. In particular, boys seemed slightly better at not negatively evaluating their emotional experiences and at coping with them than girls (i.e., we found significant but weak associations of the appraisal subscale score with sex). However, regarding the ability to tolerate negative emotions (tolerance subscale scores), feeling overwhelmed by their own emotions (absorption subscale scores) and the ability to engage in emotion regulation strategies (regulation subscale scores), no differences were found between boys and girls. This result is partially consistent with previous studies, in either youth or adult samples, in which females reported lower levels of distress tolerance than males [1,38,50,68]. More studies are needed to explore this potential association.

Aim 4. The DTS was sensitive to therapeutic change. To assess it, a subsample of children with emotional disorders who benefited from the UP-C was used. The UP-C, a transdiagnostic CBT intervention consisting of 15 weekly sessions of 90 min, was administered in group settings (groups of four to eight children and parents) by psychologists with clinical experience in CBT and training in the UP-C. The session content includes psychoeducation on emotions and their adaptive role, emotional regulation strategies to facilitate the identification and tolerance of emotional states and cognitive and behavioral strategies, aiming to promote emotional regulation across a broad spectrum of emotions [53]. After participating in the UP-C, children presented significantly higher levels of distress tolerance according to the DTS. Although distress tolerance is usually conceptualized as a transdiagnostic vulnerability factor that contributes to the development and/or maintenance of psychopathology (e.g., [4,5,6,7]), understanding of how one’s emotions operate and their adaptative function (through psychoeducation techniques) and learning techniques that promote emotional awareness and tolerance (for example, through present-moment awareness and non-judgmental techniques and emotional exposure techniques), is expected to contribute to improved levels of distress tolerance. Therefore, these results have important implications regarding the theoretical conceptualization of distress tolerance (which can then be understood as a modifiable transdiagnostic process) and clinical implications, emphasizing the importance of transdiagnostic interventions.

### 4.1. Study Novelty and Relevance

The present study is particularly relevant and novel, since it contributes to the expansion of research on the assessment of distress tolerance using a self-report questionnaire; notably, it is the first European study to do so.

There is a scarcity of psychometric measures to assess the construct of distress tolerance, which has been identified in the literature over the past few decades as an important factor regarding vulnerability to and the maintenance of psychopathology. Recently, with the emergence of transdiagnostic approaches, distress tolerance is viewed as an important transdiagnostic factor underlying psychopathology; namely, emotional disorders (e.g., [8,69]), including in childhood [25,50]. Therefore, assessing this construct through a self-report questionnaire such as the DTS allows us to enhance empirical research on the role of distress tolerance in the maintenance and vulnerability of childhood psychopathology.

Furthermore, although the DTS was developed to assess this construct in adults and was later adapted for use in children and adolescents, studies evaluating its psychometric properties are rare, and such studies have only been conducted in Chinese and American populations. Additionally, the factor structures reported by previous studies have not been consistent, and only Tonarely and Ehrenreich-May [50] included a clinical sample. Therefore, evaluating the use of the DTS in other cultures and populations (namely, in Europe) is essential to address these gaps. In particular, studying the psychometric properties of the DTS among children in a European country (such as Portugal), and especially in a clinical sample (of children with emotional disorders), is novel and relevant.

Specifically, there is no measure to assess distress tolerance in the Portuguese population, which limits empirical investigations using this critical construct; therefore, this study is of particular empirical and clinical relevance for the Portuguese population.

Additionally, the present study demonstrated that the DTS is sensitive to change. This finding can facilitate clinical evaluations of the impact of CBT interventions on distress tolerance levels (which may be an important mechanism of change). Thus, the present study may facilitate clinical trials with Portuguese children in which distress tolerance is assessed, which have not been conducted in Europe.

The DTS is a relatively short questionnaire consisting of 15 self-report items; this is an advantage, as it facilitates the quick administration of this scale, which provides advantages for applications in both clinical and research contexts.

Furthermore, this study sheds light on certain aspects that may be crucial in the examination of distress tolerance, specifically potential sex and age differences and the relationship of DTS scores with clinical symptomatology, among other factors, which may inspire further research.

### 4.2. Study Limitations

Despite these contributions, this study also has some limitations that should be noted to inform future studies. In the present study, the sample may not have been fully representative of the Portuguese population, as it was collected in only the central region of the country. Because the clinical sample of children was collected in a therapeutic setting and a change in behavioral avoidance was expected, and because participants in the community sample only completed the questionnaire once, the test–retest reliability of the DTS could not be examined. Finally, the convergent validity of the scales was not assessed using other distress tolerance measures or subscales (either self- or other-reported) or even observational/behavioral measures.

## Figures and Tables

**Table 1:** Children’s sociodemographic and clinical characteristics.

	*n* = 153
Sociodemographic characteristics	
Sex, *n* (%)	
Female	90 (58.8%)
Male	63 (41.2%)
Education level, *n* (%)	
Kindergarten	1 (0.7%)
Primary school	77 (50.3%)
Middle school	75 (49.1%)
Clinical characteristics	
Primary diagnosis, *n* (%)	
Social phobia/performance anxiety	42 (27.5%)
Generalized anxiety disorder	29 (19.0%)
Specific phobia	27 (17.6%)
Separation anxiety disorder	16 (10.5%)
Depression	12 (7.8%)
Obsessive compulsive disorder	9 (5.9%)
Anxiety disorder not otherwise specified	6 (3.9%)
Illness anxiety disorder	4 (2.6%)
Panic disorder	4 (2.6%)
Post-traumatic stress disorder	2 (1.3%)
Selective mutism	1 (0.7%)
Agoraphobia	1 (0.7%)
Presence of comorbidities, *n* (%)	
Yes	101 (66%)
No	59 (38.6%)

**Table 2:** Descriptive statistics, Cronbach’s alpha values, item-total correlations and Cronbach’s alpha values if item deleted.

	Item-Total Correlation	Alpha If Item Deleted	*M* (*SD*)	SE
Regulation subscale				
Cronbach’s alpha = 0.83				
Item 8	0.68	0.77	3.40 (1.41)	0.11
Item 13	0.76	0.58	3.24 (1.41)	0.11
Item 14	0.63	0.41	3.07 (1.38)	0.11
Absorption subscale				
Cronbach’s alpha = 0.78				
Item 2	0.67	0.66	2.95 (1.34)	0.11
Item 4	0.54	0.80	2.86 (1.44)	0.12
Item 15	0.66	0.66	3.00 (1.42)	0.12
Tolerance subscale				
Cronbach’s alpha = 0.73				
Item 1	0.65	0.53	3.07 (1.38)	0.11
Item 3	0.56	0.64	2.90 (1.33)	0.11
Item 5	0.46	0.76	2.97 (1.46)	0.12
Appraisal subscale				
Cronbach’s alpha = 0.70				
Item 6	0.22	0.72	3.30 (1.35)	0.11
Item 7	0.36	0.68	3.24 (1.53)	0.12
Item 9	0.52	0.63	3.15 (1.34)	0.11
Item 10	0.56	0.62	3.16 (1.33)	0.11
Item 11	0.52	0.63	2.40 (1.46)	0.12
Item 12	0.43	0.66	2.86 (1.51)	0.12
Total score Cronbach’s alpha = 0.89				

**Table 3:** Confirmatory factor analysis results.

	χ^2^(df)	CFI	TLI	RMSEA	SRMR	AIC
Model 1	χ^2^(86) = 144.54, *p* < 0.001	0.933	0.919	0.067	0.057	242.54
Model 2	χ^2^(86) = 141.97, *p* < 0.001	0.934	0.917	0.067	0.056	213.97
Model 3	χ^2^(86) = 143.82, *p* < 0.001	0.934	0.920	0.067	0.056	211.82
Model 4	χ^2^(87) = 150.48, *p* < 0.001	0.928	0.913	0.069	0.057	216.48
Model 5	χ^2^(86) = 144.54, *p* < 0.001	0.934	0.919	0.067	0.057	212.54

**Table 4:** Pearson correlation coefficients of DTS scores with measures of anxiety and depression (RCADS scores) and of positive and negative affect (PANAS-C-SF scores).

	DTS				
Variable	Total Score	Tolerance	Appraisal	Absorption	Regulation
1. RCADS score	−0.48 **^,a^	−0.35 **^,b^	−00.47 **^,a^	−0.48 **^,a^	−0.24 **^,b^
2. PANAS-C-SF negative affect score	−0.45 **^,a^	−0.42 **^,a^	−00.43 **^,a^	−0.43 **^,a^	−0.20 *^,b^
3. PANAS-C-SF positive affect score	0.26 **^,b^	0.21 **^,b^	0.30 **^,b^	0.33 **^,b^	0.02 ^b^

Note. ** *p* < 0.05; * *p* < 0.05; ^a^ SE = 0.07; ^b^ SE = 0.08.

**Table 5:** Correlation matrix of DTS subscales and sex/age.

DTS	Sex	Age
Total score	−0.16 *^,a^	−0.05 ^a^
Tolerance	−0.11 ^a^	−0.05 ^a^
Appraisal	−0.19 *^,a^	−0.04 ^a^
Absorption	−0.13 ^a^	−0.11 ^a^
Regulation	−0.08 ^a^	0.04 ^a^

Note. *n* = 153; * *p* < 0.05. ^a^ SE = 0.08.

**Table 6:** Differences between DTS scores at pretreatment and post-treatment.

	*M* (*SD*)	*t* Value	*p*	Cohen’s *d*	SE
DTS total score at pretreatment	2.89 (0.84)	−6.81	<0.001	0.94	0.62
DTS total score at posttreatment	3.72 (0.92)				

Note. *n* = 73 (participants who received the UP-C intervention and who completed the pre- and post-treatment measures).

## Data Availability

Data is contained within the article.

## Appendix A

**Table A1:** Content of the Portuguese version of the DTS.

Instruction	Original/English (E): Think of times that you feel distressed or upset. Select the item from the menu that best describes your beliefs about feeling distressed or upset. Portuguese Version (PT): Pensa nas vezes em que sentes emoções negativas (por exemplo, muito nervoso, ansioso, irritado, preocupado ou triste). Coloca um círculo à volta do item que melhor descreve o que pensas sobre sentires emoções negativas. Por favor responde em relação às tuas emoções negativas ‘no geral’, ou seja, em média.
Item *n*	
1	(E) Feeling distressed or upset is unbearable to me | (PT) Sentir emoções negativas é insuportável para mim.
2	(E) When I feel distressed or upset, all I can think about is how bad I feel | (PT) Quando sinto emoções negativas, eu só consigo pensar no quão mal me sinto.
3	(E) I can’t handle feeling distressed or upset. | (PT) Eu não consigo aguentar sentir emoções negativas.
4	(E) My feelings of distress are so intense that they completely Absorption take over | (PT) As minhas emoções negativas são tão intensas que tomam conta de mim / que assumem o controlo sobre mim.
5	(E) There’s nothing worse than feeling distressed or upset | (PT) Não há nada pior do que sentir emoções negativas.
6	(E) I can tolerate being distressed or upset as well as most people | (PT) Eu consigo tolerar sentir emoções negativas tão bem quanto a maioria das pessoas.
7	(E) My feelings of distress or being upset are not acceptable | (PT) Eu não devia sentir emoções negativas.
8	(E) I’ll do anything to avoid feeling distressed or upset | (PT) Eu faço tudo para evitar sentir emoções negativas.
9	(E) Other people seem to be able to tolerate feeling distressed or upset better than I can. | (PT) As outras pessoas parecem ser capazes de tolerar sentir emoções negativas melhor do que eu.
10	(E) Being distressed or upset is always a major ordeal for me. | (PT) Sentir emoções negativas é sempre um enorme desafio para mim.
11	(E) I am ashamed of myself when I feel distressed or upset. | (PT) Eu tenho vergonha de mim mesmo quando sinto emoções negativas.
12	(E) My feelings of distress or being upset scare me. | (PT) As minhas emoções negativas assustam-me.
13	(E) I’ll do anything to stop feeling distressed or upset. | (PT) Eu faço tudo para deixar de sentir emoções negativas.
14	(E) When I feel distressed or upset, I must do something about it immediately. | (PT) Quando sinto emoções negativas, eu tenho que fazer algo para controlar isso imediatamente.
15	(E) When I feel distressed or upset, I cannot help but concentrate on how bad the distress actually feels. | (PT) Quando sinto emoções negativas, eu não consigo deixar de pensar no quão mau é sentir-me assim.
